# Brain Injury Awareness Month — March 2013

**Published:** 2013-03-15

March is Brain Injury Awareness Month. Through scientific research, programs, and education, CDC works to prevent traumatic brain injury (TBI) from all causes and ensure that TBI survivors receive optimal care.

A TBI, whether caused by a fall in the home or on a playground, a car crash, or by being struck by an object or another person, can disrupt the normal functions of the brain. TBIs, which range from mild concussions to severe, life-threatening injuries, can be prevented.

Research indicates that in the United States, 1) males have the highest rates of TBI; 2) the youngest children and older adults are at highest risk for sustaining fall-related TBIs; 3) adolescents and young adults (i.e., persons aged 15–24 years) have the highest rates of motor vehicle–related TBIs; and 4) adults aged ≥75 years have the highest rates of TBI-related hospitalization and are more likely to die from TBI (either TBI alone or along with other injuries or illnesses) than any other age group (1).

The burden of TBI can be reduced through primary prevention strategies and improvements in the health and quality of life for TBI survivors. CDC recommends integrating public health prevention and health-care delivery systems, including efficient, effective care and rehabilitation services to address the issue of TBI among at-risk populations. Additional information about TBI management is available at http://www.cdc.gov/traumaticbraininjury, information about preventing motor vehicle–related TBIs is available at http://www.cdc.gov/motorvehiclesafety, and information about preventing fall-related TBIs is available at http://www.cdc.gov/homeandrecreationalsafety/falls.
